# Moyamoya Syndrome in a Patient With Klippel-Trenaunay Syndrome

**DOI:** 10.7759/cureus.11906

**Published:** 2020-12-04

**Authors:** Cameron Brimley, Christoph J Griessenauer, Clemens M Schirmer, Oded Goren

**Affiliations:** 1 Neurosurgery, Geisinger Health System, Danville, USA; 2 Neurosurgery, Geisinger Neuroscience Institute, Geisinger Medical Center, Danville, USA

**Keywords:** klippel trenaunay syndrome, moyamoya syndrome, sta mca bypass

## Abstract

Moyamoya syndrome consists of internal carotid artery stenosis with development of collateral vasculature responsible for ischemic events and cerebral hemorrhage. Moyamoya vasculopathy is commonly treated with external carotid artery to internal carotid artery bypass, either through direct or indirect anastomosis. Klippel-Trenaunay Syndrome (KTS) is a tissue hyper-proliferation disorder known to have a significant angio-dysplastic component to the pathology. No other instances of a patient with both KTS and Moyamoya syndrome are presently reported in the literature.

We present a patient who had been diagnosed with KTS as a child who was found to have Moyamoya vasculopathy after experiencing frequent cerebral ischemic events. He underwent a left direct superficial temporal artery to middle cerebral artery bypass with subsequent significant improvement of his stroke symptoms.

This case report demonstrates an association between KTS and Moyamoya syndrome with a possible shared pathophysiology. Patients with KTS may benefit from screening for cerebral ischemic events and monitoring for development of Moyamoya syndrome.

## Introduction

Moyamoya disease and Moyamoya syndrome are cerebrovascular disorders with a pathophysiological basis of blood vessel intima smooth muscle proliferation. This results in progressive stenosis of the distal aspects of the bilateral internal carotid arteries and their proximal branches, resulting in reduced blood flow in the major anterior circulation vessels. This leads to compensatory development of collateral vasculature from vessels near the carotid apex, cortical surface, leptomeninges, and external carotid arteries. Patients with this vasculopathy who also have a relevant associated condition or risk factors are categorized as having Moyamoya syndrome, while those with no known associated risk factors have Moyamoya disease. Patients with unilateral vasculopathy are also considered to have Moyamoya syndrome, even if no other risk factors are identified. In children diagnosed with Moyamoya syndrome, the underlying disease is typically congenital and includes sickle cell disease, neurofibromatosis type 1, and Down’s syndrome. Adults with Moyamoya syndrome typically have an acquired pathology [1].

The natural history of Moyamoya vasculopathy typically involves ischemic strokes in children while adults more commonly experience hemorrhagic strokes. Direct and indirect bypass surgery helps reduce the risk of additional cerebral ischemic and hemorrhagic events by augmenting cerebral perfusion, reducing hypoxia, and facilitating regression of the Moyamoya vessels [2].

Klippel-Trenaunay Syndrome (KTS) is a blood vessel, soft tissue, and bone hyper-proliferation disorder. Patients present with port-wine stains (cutaneous hemangiomata), venous malformations with a few case reports of cavernous malformations, overgrowth of soft tissue/bones, and lymphedema, typically localized to one limb [3]. We present a case of a patient with both KTS and Moyamoya syndrome and discuss a possible common pathophysiologic origin. There are no reported cases in the literature about patients with KTS also diagnosed with Moyamoya syndrome.

## Case presentation

A 29-year-old male was referred to our neurovascular team after experiencing transient ischemic attacks, increasing in frequency and severity over the past 13 years, particularly worsening over the past three months. He was primarily experiencing staring spells accompanied by a right facial droop and slurred speech, but also noted that he was having episodes of right-hand weakness and decreased coordination. His was noted to have left lower extremity lymphedema with limb enlargement and varicosities that started as a child, which had progressed over the past 20 years. He had chronic headaches, but had never had seizure activity. Based on the clinical presentation, he was diagnosed with Klippel-Trenaunay Syndrome by his pediatrician. As part of the Klippel-Trenauna Syndrome with the lymphedema, he was frequently on antibiotics for cellulitis of his left leg, but denied any prior symptoms or issues with his right arm or leg. Additional significant medical history included hypertension, hyperlipidemia, and obesity.

Initial imaging with a CT scan of his head showed multiple hypodense areas within the left frontal and parietal lobes. He was seen by his neurologist who obtained a brain MRI showing left frontal and parietal areas of T2 hyperintensity concerning for watershed infarcts. A magnetic resonance angiography (MRA) showed severe left and mild right distal internal carotid artery (ICA) and proximal middle cerebral artery (MCA) stenosis. He was started on daily Aspirin and came to our clinic for assessment where it was recommended he undergo digital subtraction angiography.

**Figure 1:**
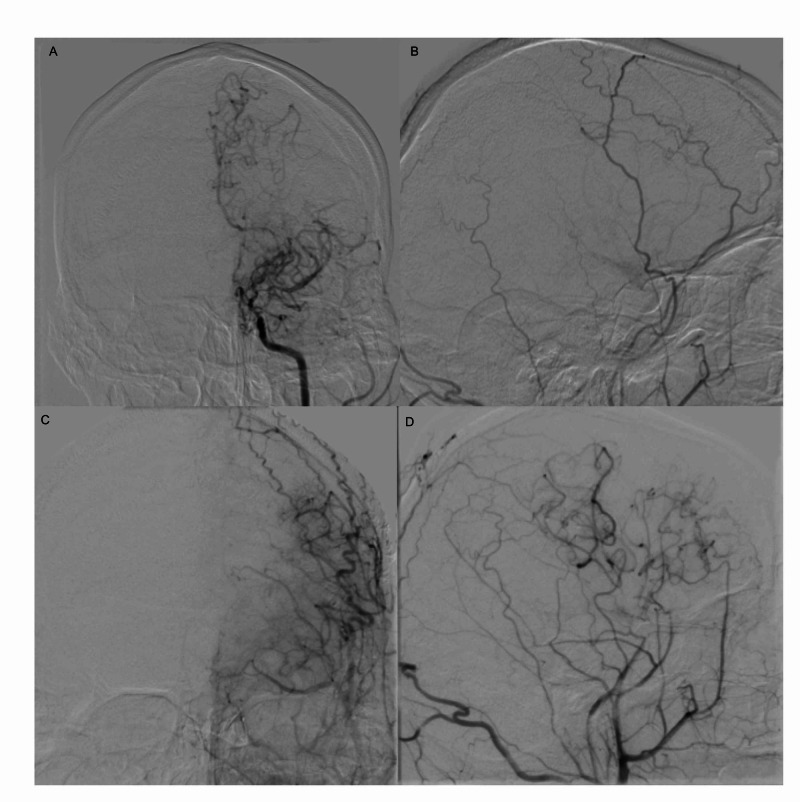
Pre- and post-bypass digital subtraction angiography (A) Pre-bypass anterior-posterior digital subtraction angiogram (DSA) of the left internal carotid artery demonstrating severe, flow-limiting distal internal carotid artery (ICA) and proximal middle cerebral artery (MCA) stenosis with the formation of many Moyamoya vessels. (B) Pre-bypass lateral DSA of the external carotid artery. (C) One year post-bypass anterior-posterior and (D) lateral DSA of the left external carotid artery demonstrating expected vascular proliferation.

The angiogram confirmed the findings of the MRA, specifically identifying moderate narrowing of his left internal carotid artery at the carotid terminus, severe flow limiting stenosis at the proximal M1 segment of the left MCA with an abundance of Moyamoya vessels. The right internal carotid artery demonstrated mild, non-flow limiting arterial caliber narrowing at the carotid terminus with opacified Moyamoya vessels (Figure 1A, 1B).

**Figure 2:**
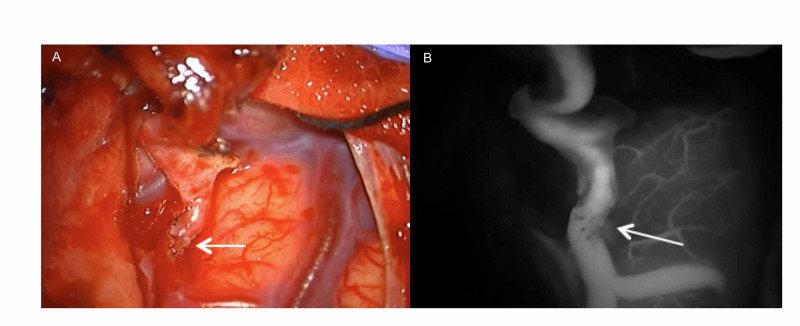
Intra-operative images (A) Microscope intra-operative view (Arrow indicating the junction of the left STA-MCA bypass). (B) Intraoperative indocyanine green angiography documenting the patency of the anastomosis (Arrow indicating the junction of the left STA-MCA bypass). STA: Superficial temporal artery; MCA: Middle cerebral artery.

Based on these findings and the recurrent watershed infarcts occurring with increasing frequency, an external carotid to internal carotid bypass surgery was discussed with the patient who elected to proceed.There were no complications with the surgery. The indocyanine green (ICG) run and intraoperative angiogram confirmed patency of the bypass (Figure 2). At each of his three follow-up visits, he continued to experience improvement of his symptoms without any recurrent ischemic events.

He was seen in follow-up at one year after surgery where he had no neurological deficits and noted his transient ischemic attacks had resolved completely. Follow-up digital subtraction angiography was performed showing significant radiographic vascular proliferation from the bypass site with some regression of Moyamoya vasculature, Matsushima Grade B (Figure 1C, 1D).

## Discussion

Klippel-Trenaunay Syndrome is a disease of tissue hyper-proliferation secondary to an aberrant gain of function mutation of the PIK3CA gene, phosphatidylinositol 3-kinase subunit with an incidence of approximately 1:100,000 [3]. Some vascular conditions associated with KTS include hemangiomas and venous varicosities with a few reported cases of associated cavernous malformations [3-5]. Examples include a 64-year-old male with KTS who also had multiple cavernomas in his brain and spinal cord, a 23-year-old female with KTS was found to have a cavernoma within her spinal cord [3], and another patient with KTS was diagnosed with bilateral ICA occlusions and giant P1 aneurysm [4]. Moyamoya syndrome is defined as a distal ICA and proximal branch stenosis inducing formation of collateral accessory vasculature in the setting of another underlying condition [1-2]. No known cases of KTS associated with Moyamoya syndrome are published. With both KTS and Moyamoya present in this patient, the pathophysiology underlying KTS may conceivably be implicated in the formation of his Moyamoya syndrome. The gain of function mutation of KTS with resultant tissue hyper-proliferation may play a role in hyperplasia of the terminal ICA intima, contributing to stenosis and subsequent accessory vasculature formation.

Alternatively, considering that this patient’s BMI was greater than 60, the development of his Moyamoya vasculopathy could also have been secondary to progressive intracranial atherosclerosis, causing distal ICA and proximal MCA stenosis and subsequent lenticulostriate collateralization. This pathogenesis would be in contrast to a direct effect of the pre-existing KTS mutation. Interestingly, other areas of severe atherosclerosis were not clearly evident on pre-operative vascular imaging with the most significant disease in the distal internal carotid, making an atherosclerosis cause less likely.Additional workup for alternative causes of vasculitis/vasculopathy was not pursued prior to the direct bypass, which is a limiting factor in more definitively establishing an association. A lumbar puncture, vessel biopsy, or vessel wall imaging could aid in establishing a vasculitis or the presence of atherosclerosis and potentially an alternative cause behind this patient’s Moyamoya vasculopathy. In either scenario, these findings indicate that patients with KTS likely have a potential higher than average risk of developing Moyamoya.

Identifying patients with KTS who have also developed Moyamoya concurrently in a spontaneous fashion would be unlikely as the incidence of both diseases is very low, approximately 1:100,000 for either disease. Both diseases occurring coincidently would have around a 1:100,000,000 chance. With the incidence of either disease as well as the calculated incidence of both disease occurring together extremely low, and with this being the first case report with both pathologies, it is difficult to clearly determine if the presence of KTS increases the risk of developing Moyamoya. However, if KTS, either because of the hyperproliferation mutation or the secondary effect of atherosclerosis from obesity does increase the risk of acquiring Moyamoya vasculopathy, additional screening of KTS patients for Moyamoya vasculopathy may be worthwhile.

This case also demonstrates the effective use of direct superficial temporal artery to middle cerebral artery bypass in a patient with Moyamoya syndrome. The post-operative follow-up angiogram shows radiographic vascular proliferation, the patient’s symptoms resolved, and he experienced an improvement in quality of life.

## Conclusions

Moyamoya syndrome is associated with a significant risk of ischemic stroke and intracerebral hemorrhage and is typically progressive in nature. Patients with sickle cell disease, neurofibromatosis type 1, and Down’s syndrome are known to be at risk of developing Moyamoya vasculopathy, but through this case, we also demonstrate that patients with Klippel-Trenaunay syndrome may also be at a higher risk and represent the first reported case of a possible relation between these vasculopathic and hyperproliferative disorders. Awareness of a possible relationship between these two syndromes may lead to more prompt and effective treatment.
